# Impact of Climate Variability and Abundance of Mosquitoes on Dengue Transmission in Central Vietnam

**DOI:** 10.3390/ijerph17072453

**Published:** 2020-04-03

**Authors:** Luong Thi Nguyen, Huy Xuan Le, Dong Thanh Nguyen, Ha Quang Ho, Ting-Wu Chuang

**Affiliations:** 1 International Master/PhD Program in Medicine, College of Medicine, Taipei Medical University, 110 Taipei, Taiwan; m142106004@tmu.edu.tw; 2 Department of Tropical Diseases, Baichay Hospital, 57000 Quang Ninh, Vietnam; 3 Nha Trang Pasteur Institute, 01000 Khanh Hoa, Vietnam; lexuanhuy75@yahoo.com.vn; 4 Department of Vector Control and Border Quarantine, Nha Trang Pasteur Institute, 01000 Khanh Hoa, Vietnam; dongpasteur@gmail.com (D.T.N.); hoquangha39@yahoo.com (H.Q.H.); 5 Department of Molecular Parasitology and Tropical Diseases, School of Medicine, College of Medicine, Taipei Medical University, 110 Taipei, Taiwan

**Keywords:** dengue fever, central Vietnam, Indian Ocean Dipole, El Niño-Southern Oscillation index, vector index

## Abstract

Dengue fever is an important arboviral disease in many countries. Its incidence has increased during the last decade in central Vietnam. Most dengue studies in Vietnam focused on the northern area (Hanoi) and southern regions but not on central Vietnam. Dengue transmission dynamics and relevant environmental risk factors in central Vietnam are not understood. This study aimed to evaluate spatiotemporal patterns of dengue fever in central Vietnam and effects of climatic factors and abundance of mosquitoes on its transmission. Dengue and mosquito surveillance data were obtained from the Department of Vector Control and Border Quarantine at Nha Trang Pasteur Institute. Geographic Information System and satellite remote sensing techniques were used to perform spatiotemporal analyses and to develop climate models using generalized additive models. During 2005–2018, 230,458 dengue cases were reported in central Vietnam. Da Nang and Khanh Hoa were two major hotspots in the study area. The final models indicated the important role of Indian Ocean Dipole, multivariate El Niño-Southern Oscillation index, and vector index in dengue transmission in both regions. Regional climatic variables and mosquito population may drive dengue transmission in central Vietnam. These findings provide important information for developing an early dengue warning system in central Vietnam.

## 1. Introduction

Dengue fever is an *arboviral infection* caused by the dengue virus and has four antigenically distinct serotypes (DENV 1–4) [1]. The dengue virus is transmitted from an infected person to a susceptible one through the bite of *Aedes aegypti* (principal vector) or *Aedes albopictus* mosquito [2]. Dengue fever circulates mainly in tropical and subtropical regions, although outbreaks have been reported in temperate regions [3,4]. Approximately half of the global population lives in countries where dengue is an endemic [5]. Dengue fever includes a wide spectrum of illnesses, ranging from asymptomatic to severe. Severe dengue is characterized by plasma leakage, severe hemorrhage, and severe organ failure, possibly leading to dengue hemorrhagic fever or dengue shock syndrome [6]. Secondary infection with a heterogeneous dengue serotype mediated by antibody-dependent enhancement is a putative risk factor for severe dengue and may be fatal without appropriate treatment [7,8].

Dengue transmission varies over space and time and is influenced by multiple factors [9,10]. Climate variables are considered major contributors to the spread of dengue [11]. Rainfall provides a water source that serves as a breeding site for female mosquitoes and an environment for immature larva/pupa, resulting in abundant mosquitoes and dengue outbreaks [11,12]. However, a large amount of rain may have a flushing effect, increasing the mortality rate of adult mosquitoes and larvae [13,14]. Temperature plays a major role in the development, survival, and feeding behavior of mosquitoes and encourages viral replication inside the vector [11]. Higher temperature shortens the gonotrophic cycle and extrinsic incubation period [15,16]. Conversely, extreme temperature negatively impacts the vector population by increasing egg and adult mosquito mortality and by reducing the eclosion rate [17].

At the regional level, global warming, resulting from climate change, may expand the regions with suitable conditions for vector-borne disease transmission [18]. Two coupled ocean-atmosphere phenomena, i.e., the El Niño-Southern Oscillation (ENSO) and Indian Ocean Dipole (IOD), have been studied to determine their roles in the global reemergence of certain infectious diseases [19,20]. ENSO and IOD have shown significant correlations with local climatic variability in many regions, especially temperature and precipitation [21,22,23]. ENSO and IOD combine with local weather parameters to drive the dynamics of mosquito-borne diseases including malaria and dengue fever [23,24,25].

Vector abundance is another trigger for dengue outbreaks [26]. Some studies have reported a positive correlation between mosquito population and dengue cases [27,28]. However, studies conducted in Malaysia and Colombia did not show any relationship between vector abundance and the incidence of dengue [29,30,31]. Although the influence of the mosquito index on dengue transmission is variable, regular mosquito surveillance remains important and is included in the dengue control programs implemented in many epidemic or endemic countries [31,32,33].

Dengue fever is hyperendemic in Vietnam, where cocirculation of multiple DENV serotypes has been identified, especially in southern Vietnam [34,35]. Vietnam is among the five Asian-Pacific countries with the heaviest dengue burden [2]. During 1980–2010, Vietnam reported nearly 2.5 million cases and six major outbreaks [36]. Most dengue studies conducted in Vietnam focused on the northern area (Hanoi) and southern regions [34,37,38,39]. The central region of Vietnam is also an endemic area for dengue; however, there is little relevant research in this region [40,41]. Therefore, dengue transmission dynamics and relevant environmental risk factors in central Vietnam are not understood.

This study aimed to fill the knowledge gap concerning dengue transmission in central Vietnam by investigating the spatiotemporal distributions of dengue outbreaks and evaluating the impact of climate and the vector index on dengue transmission in this region. Our hypotheses were as follows: 1) dengue fever outbreaks in central Vietnam might not occur randomly in space and time, and 2) seasonality of dengue transmission in central Vietnam might be associated with certain climate factors and vector abundance.

## 2. Materials and Methods

### 2.1. Study Area

**Figure 1:**
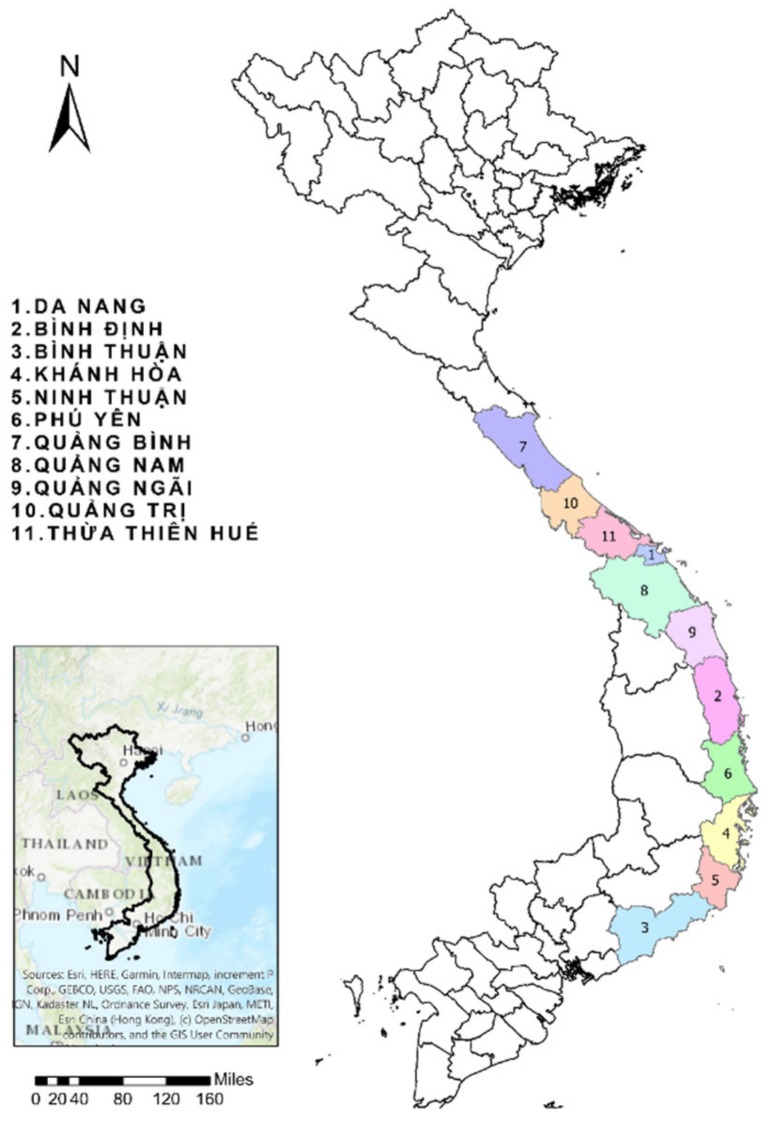
The eleven provinces in central Vietnam included in this study.

This study included 11 coastal provinces within the central area of Vietnam, in which all information on dengue is managed by the Nha Trang Pasteur Institute (Figure 1). The study area has a population of 11,898,300, with an average population density of approximately 207/km^2^. The lowest population density is in Quang Binh (110/km^2^) and the highest is in Da Nang (814/km^2^). The climate in central Vietnam can be divided into two zones: the north-central coast and the south-central coast [42]. The north-central coast has a cold dry winter season, lasting from December to March, and a rainy season, with the highest rainfall recorded in October [42]. Conversely, the south-central coast has a tropical monsoon climate with a rainy season and a dry season. The rainy season normally lasts from September to December, with the highest rainfall in October and November, and the dry season lasts from January to April [42].

### 2.2. Dengue Case Data

**Figure A1:**
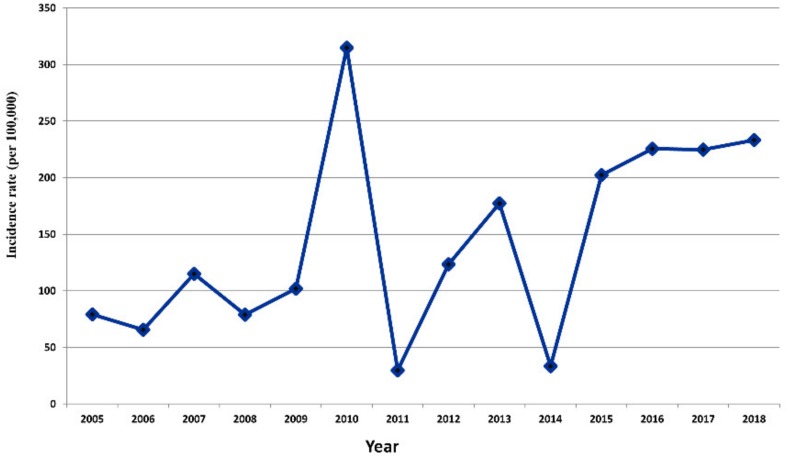
Annual dengue incidence (per 100,000) in central Vietnam between 2005 and 2018.

Data of confirmed dengue cases in the 11 central provinces of Vietnam were obtained from the Department of Vector Control and Border Quarantine at the Nha Trang Pasteur Institute. Overall, the annual incidence of dengue in central Vietnam showed significant fluctuations between 2005 and 2018 but has remained high since 2015 (Figure A1). Given that the spatiotemporal resolution of the data has steadily improved in the past decade, we analyzed the incidence of dengue in different temporal periods. We visualized spatial patterns for the incidence of dengue between 2011 and 2018 and used the monthly cases of dengue, which have been available since 2014, for our climate model.

A dengue case was defined, according to the guideline of the Vietnam Ministry of Health, as a positive serological test result for immunoglobulin M (IgM) or nonstructure protein 1 (NS1) in enzyme-linked immunosorbent assay, nucleotide sequence (polymerase chain reaction), or isolation of the virus [43].

The population data in each province were obtained directly from the General Statistics Office of Vietnam (gso.gov.vn). The research protocol was approved by the Taipei Medical University-Joint Institutional Review Board (No. 201602014), and all data were analyzed anonymously.

### 2.3. Mosquito Data

Data on the abundance of mosquitoes were obtained from a monthly mosquito survey from provincial preventive health centers and were sent to the Department of Vector Control and Border Quarantine at the Nha Trang Pasteur Institute. The vector indices used in this study were density index (DI, mean number of female *Aedes* mosquitoes per house inspected), house infestation index (HIF, percentage of houses infested with adult *Aedes* female mosquitoes), container index (CI, percentage of water-holding containers infested with active immature larvae), Breteau index (BI, number of positive containers per 100 houses inspected), and house index (HI, percentage of houses infested with larvae and/or pupa). Monthly mosquito indices were available for 2015–2018.

### 2.4. Climate Data

Weather stations are not widely available in central Vietnam. Therefore, we acquired climate data including land surface temperature (LST) and rainfall from satellite images as proxies. Previous studies have shown high correlations between ground-level measurements and satellite-derived parameters [44,45]. We obtained LST data using a moderate resolution imaging spectroradiometer (MODIS), which is a key instrument aboard the Terra and Aqua satellites (lpdaac.usgs.gov). The processes used to obtain satellite images and to estimate LST have been well documented [25,46]. Average monthly daytime, nighttime, and mean temperatures were calculated from 8-day composite images acquired using the MODIS. The total monthly rainfall variables were derived from global precipitation measurement data (pmm.nasa.gov/GPM). Local climate parameters were summarized at the province level for further analysis. For regional climatic phenomena, we used the multivariate ENSO index (MEI) to quantify the effects of ENSO. MEI is a composite of six significant variables observed in the tropical Pacific Ocean [47]. The monthly dipole mode index (DMI) which reflects the sea surface temperature in the Indian Ocean was used to evaluate the influence of IOD [22]. The associations between the incidence of dengue and regional climatic phenomena have been well described elsewhere [25].

### 2.5. Statistical Analysis

Understanding the spatiotemporal distribution of a dengue outbreak is important in terms of localizing interventions to control the disease. Geographic Information System techniques provide useful opportunities to study the dynamics of dengue transmission [48,49,50]. We visualized the spatiotemporal patterns of dengue in central Vietnam using ArcGIS 10.4 (ESRI, Redland, CA, USA).

The associations of climatic variations and vector abundance on the incidence of dengue were analyzed using a generalized additive model (GAM). This model has been applied in many vector-borne disease studies and uses a spline smoothing operator to fit data with nonlinear relationships [51,52,53,54]. A negative binomial distribution was assumed in the GAM analysis [55] to deal with the overdispersion issue caused by the high variability in the dengue incidence data. We selected Da Nang and Khanh Hoa, the two major dengue hotspots in central Vietnam, to build the climate models. Daytime temperature, nighttime temperature, rainfall, regional climatic phenomena (MEI and IOD), and mosquito indices up to 3 lag months were included as factors affecting the incidence of dengue. Assuming that all risk factors may contribute to the dynamics of dengue transmission, we fixed all the environmental variables in the model and evaluated the different effects in the lag period using Equation (1), where *Y_t_* denotes the dengue incidence rate at month *t* in Da Nang or Khanh Hoa; *Temperature_t,k_*, *Rainfall_t,k_*, *IOD_t,k_* and *MEI_t,k_* denotes the environmental variables at month *t* with a different lag period *k* (3 ≥ *k* ≥ 0); and represents the spline smooth function. To avoid collinearity, the daytime and nighttime temperatures (DTM and NTM, respectively) were evaluated one at a time.

*Y_t_ = s(Temperature_t,k_) + s(Rainfall_t,k_) + s(IOD_t,k_) + s(MEI_t,k_)* (1)

Given that mosquito population data were available only for 2015–2018, we generated submodels to analyze the impact of the mosquito indices and regional climate variables on dengue transmission (Equation (2)). The local climate variables were not included in the submodel analysis because our results demonstrated that regional level climate variables play a more important role than local climate variables, where *Vector_t,k_* indicates the different types of monthly vector indices (i.e., the density index, house infestation index, house index, container index, and Breteau index).

*Y_t_ = s(Vector_t,k_) + s(IOD_t,k_) + s(MEI_t,k_)* (2)

The performance of the model was evaluated using Akaike’s information criterion (AIC). The smallest AIC indicates the model with the best fit [56]. Finally, we examined the importance of the different variables in the best-fitted models by calculating the difference in the AIC value (∆AIC), where a larger value indicates that the variable is more important in the model. All statistical analyses were performed using R software 3.5.1 (R Development Core Team, R Foundation for Statistical Computing, Vienna, Austria), and the mgcv package was used to develop the model.

## 3. Results

### 3.1. Dengue Transmission in Central Vietnam

**Figure 2:**
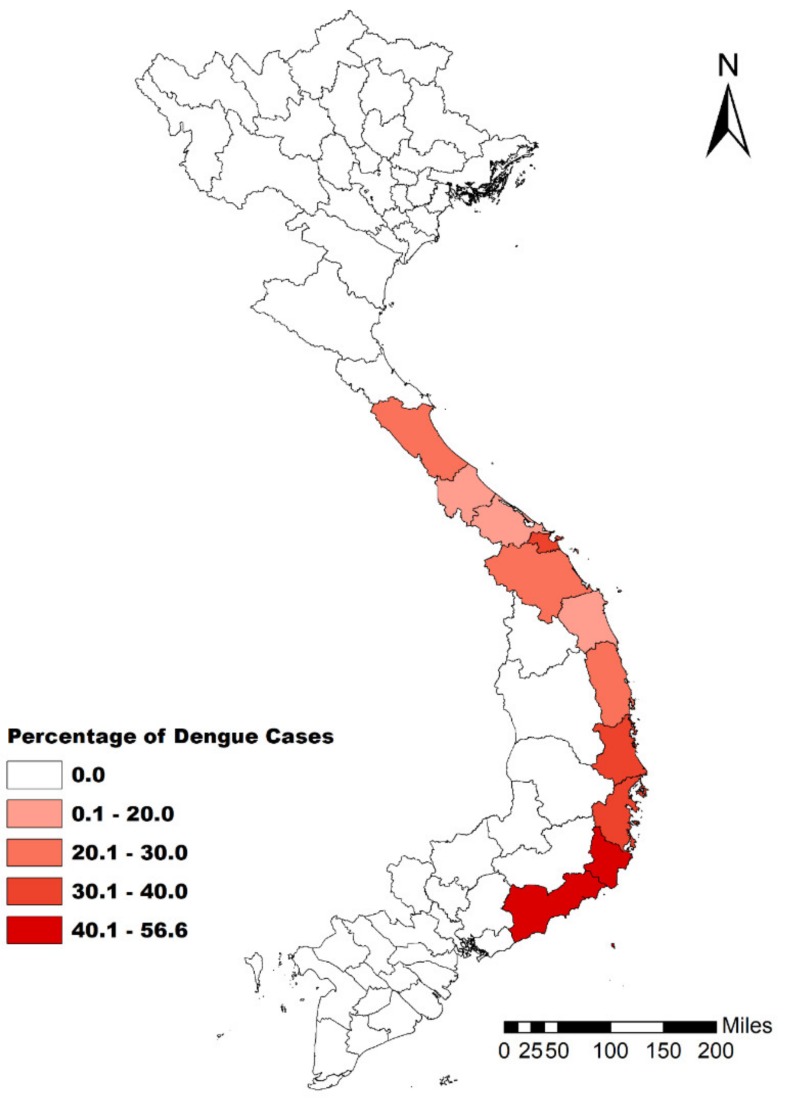
Percentage of dengue cases in central Vietnam among children under the age of 15 years.

Central Vietnam reported 230,458 dengue cases during 2005–2018; the annual number of cases ranged from 3421 to 35,865, with a median of 12,840. From 2014 to 2018, central Vietnam reported 109,628 cases with 12 deaths (mortality rate, 0.011 %). In 2014–2018, 34.22 % of all cases occurred in children aged <15 years. The percentage of cases of dengue in younger people gradually increased moving southward in the south-central coast region (Figure 2). The annual incidence of dengue in central Vietnam demonstrated wide variations after 2010, with outbreaks occurring every year after 2015 (Figure A1).

### 3.2. Spatiotemporal Patterns of Dengue Transmission in Central Vietnam

**Figure 3:**
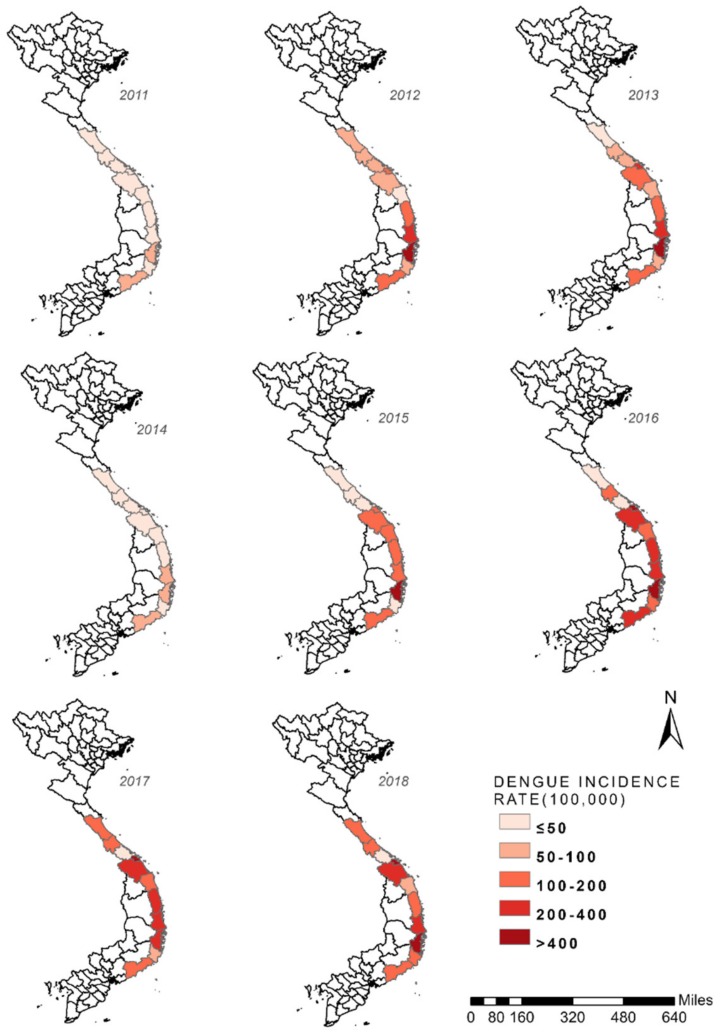
Spatial and temporal patterns in the incidence of dengue in central Vietnam between 2011 and 2018.

Figure 3 shows the spatial and temporal patterns in the incidence of dengue for 11 provinces in central Vietnam during 2011–2018. The median annual incidence of dengue was 119.2 (range, 29.8–315) cases per 100,000 population during the study period. Most cases occurred in the south-central coast region, with the highest incidence in the Khanh Hoa province. The incidence rate in Da Nang, the northern part of the coast region, increased dramatically after 2016 compared with that in the previous years. During 2011–2018, 42.1 % of dengue cases in central Vietnam occurred in these two areas. Therefore, Da Nang and Khanh Hoa were considered two dengue hotspots in central Vietnam, warranting further analysis.

### 3.3. Dengue Transmission in Da Nang and Khah Hoa

**Figure 4:**
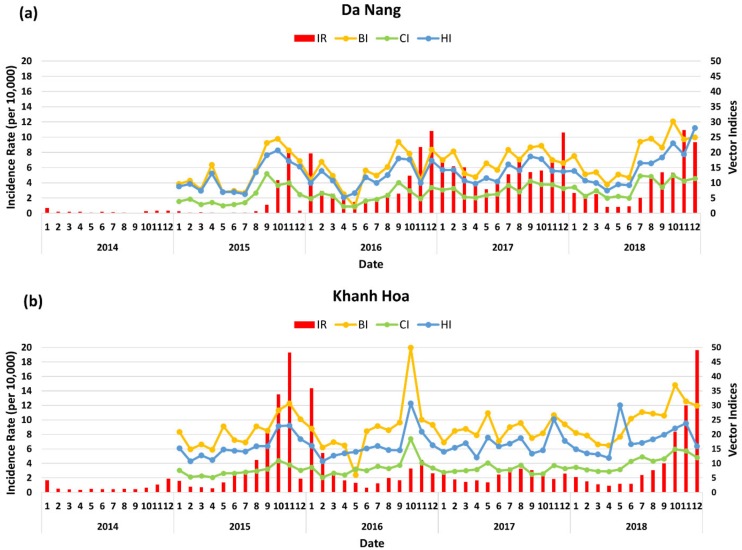
Monthly dengue incidence rate (per 10,000) and vector indices in Da Nang (**a**) and Khanh Hoa (**b**) between 2014 and 2018 (*density index (DI) and house infestation index (HIF) were excluded because DI* *had a* *very low value during the study period* *and HIF did not* *correlate* *with dengue transmission in the study areas)*. IR—incidence rate; BI—Breteau index; CI—container index; HI—house index.

In Da Nang, 25,428 dengue cases (average, 30,042 cases/10,000 population/year) were reported during 2011–2018. The incidence rates increased significantly from 2015 to 2016, decreased slightly in 2017, and then increased again in 2018 (Figure 4a). The monthly incidence rate was relatively higher in October and November, and this trend persisted until January of the following year. In 2016, a massive outbreak occurred in Da Nang, with the highest incidence occurring in December 2016. The incidence remained high throughout 2017 but decreased in the first 7 months of 2018 and then showed a steady increase to the end of 2018. In Khanh Hoa, 38,884 dengue cases (average, 39.876 cases/10,000 population/year) were reported in 2011–2018, with peaks of incidence in 2013, 2015, and 2018. The incidence of dengue in Khanh Hoa increased rapidly and peaked in 2015, decreased in 2016, remained low in 2017, and increased again in 2018 (Figure 4b). During the study period, dengue transmission in Khanh Hoa and Da Nang showed similar seasonal patterns, but the intensity of outbreaks was higher in Khanh Hoa. Overall, the values of the vector indices (BI, CI, and HI) were higher in Khanh Hoa. The seasonal patterns of vector indices were similar to dengue transmission, and the peak usually appeared before the peak of dengue outbreaks in both areas (Figure 4a,b).

### 3.4. Influence of Climate Variation and Vector Index on Dengue Transmission in Da Nang and Khanh Hoa

The temperatures in Khanh Hoa and Da Nang were similar during the study period, but Da Nang had a higher average monthly rainfall (49.86 ± 102.21 mm vs. 19.32 ± 43.35 mm; Table 1). The seasonal pattern indicated that the rainy season occurred mainly in October and November in both areas (Table A1).

**Figure 5:**
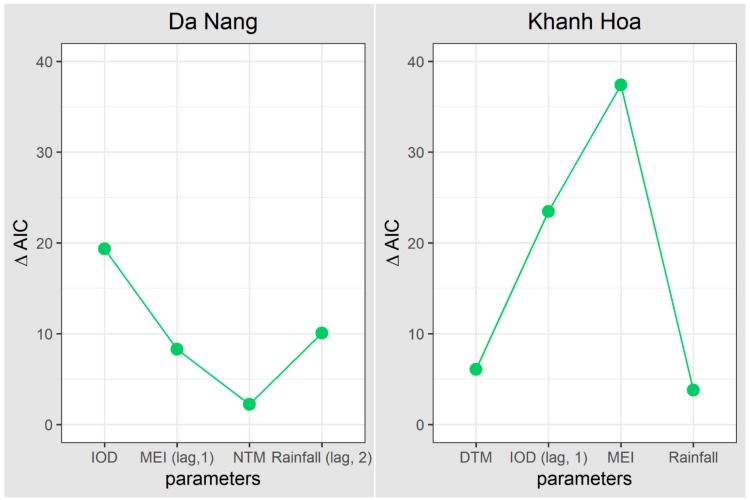
Importance of model parameters (Table 2) in Da Nang and Khanh Hoa. IOD—Indian Ocean Dipole; MEI—multivariate ENSO index; DTM—daytime temperature; NTM—nighttime temperature; AIC—Akaike information criterion.

Table 2 shows the best-fitted model for regional and local climatic variables and dengue transmission in Da Nang and Khanh Hoa. Model 1 is the full model, and the other models were used to examine the importance of temperature, rainfall, IOD, and MEI. Overall, IOD in the current month is the dominant climatic risk factor for dengue transmission (largest ∆AIC), followed by rainfall (lag = 2 months), MEI (lag = 1 month), and nighttime temperature (NTM) in Da Nang (Figure 5). In Khanh Hoa, MEI and IOD with a 1-month lag played an important role in dengue transmission, followed by daytime temperature (DTM) and rainfall.

**Figure 6:**
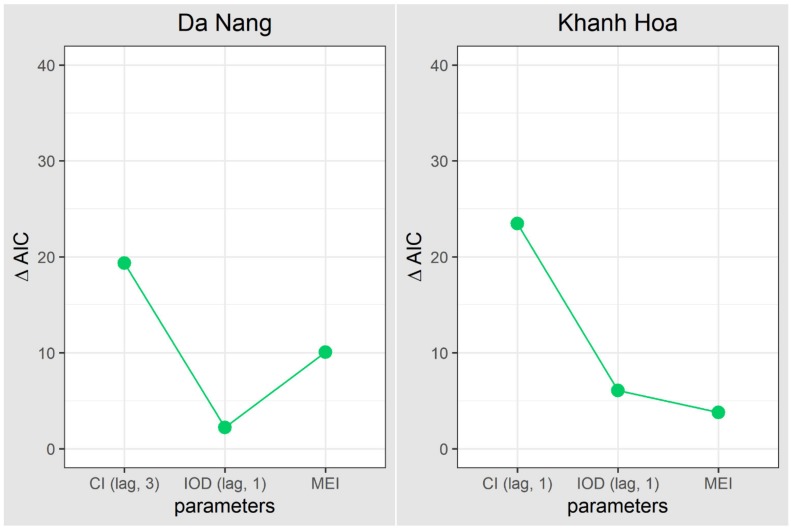
Importance of model parameters (Table 3) in Da Nang and Khanh Hoa. IOD—Indian Ocean Dipole; MEI—multivariate ENSO index; CI—Container Index; AIC—Akaike information criterion.

The sub-models included only regional climate variables and mosquito indices (Table 3) because our models indicated that regional climate phenomena had a more significant impact on dengue transmission. The best-fitted model (model 1) includes IOD (lag = 1 month), MEI in the current month, and container index (lag = 3 months in Da Nang and 1 month in Khanh Hoa). Both models for Da Nang and Khanh Hoa showed that the container index had the strongest relationship with dengue transmission, followed by MEI and IOD (Figure 6).

## 4. Discussion

This study examined the spatiotemporal patterns of dengue fever in 11 coastal provinces in central Vietnam, and its results elucidate the fundamental epidemiology of dengue outbreaks in this area. The south-central coast region has a large proportion of reported dengue cases in central Vietnam. Two hotspots for dengue transmission were identified in Da Nang and Khanh Hoa provinces. Herein, regional climate parameters were found to play an important role in dengue transmission, whereas local climatic variables had different impacts on dengue transmission depending on the region. Regional climatic factors accompanied by mosquito indices may drive dengue transmission in these two hotspots.

The risk of severe dengue varies according to the age of the host. A cohort study of laboratory-confirmed dengue infections in Nicaraguan infants, children, and adults showed that the proportion of patients who had dengue fever with warning signs or severe dengue decreased with increasing age [57]. In hyperendemic Asian countries, dengue fever is a leading cause of hospitalization and death in children [58,59]. According to a report published by the Vietnam Ministry of Health in 2015, the incidence of dengue in the younger age groups increased moving southward (unpublished data). Herein, we also observed a southward pattern in the incidence of dengue in children aged <15 years in the south-central coast region. However, the mechanism contributing to this phenomenon is still unclear due to lack of serological data. Human movement, intervention measurements, or vector ecology might explain the difference. A human mobility study in the city of Nha Trang showed that younger individuals spent more time closer to their home than those aged 15–17 years and young adults [40]. That observation suggests that children aged <15 years are at greater risk of bites by the *Aedes aegypti* mosquito. Conversely, older age groups are more likely to be exposed to outdoor mosquitoes such as *Aedes albopictus*. Furthermore, the distribution of the *Aedes aegypti* mosquito shows a southward pattern of distribution [60,61]. However, whether causality can be inferred requires further investigation.

Dengue transmission occurs mainly in the rainy season, which is during the second half of the year in Vietnam. In Hanoi, where more than 75 % of dengue cases in northern Vietnam occur, dengue fever transmission showed a seasonal pattern, with a gradual increase in incidence from April to August and a peak in September and October [38,62]. Southern Vietnam is known to be a hyperendemic area where dengue transmission occurs all year round [34,63] with a particularly high incidence from August to November [38]. In central Vietnam, there is a difference in dengue transmission between the north-central and south-central coast regions. The north-central region has a lower incidence rate, with most of the dengue cases reported in the south-central region and in two hotspots, i.e., Da Nang and Khanh Hoa. In our study, dengue transmission also showed a seasonal pattern in these two hotspots, with a high rate of transmission from October to December. The incidence of dengue increased significantly in Da Nang and Khanh Hoa during the study period. Before 2009, the respective monthly numbers of dengue cases reported in Da Nang and Khanh Hoa were usually <500 and 1200 [38]. Compared with the rates reported in 1994–2013, the annual incidence of dengue in Khanh Hoa increased by approximately 1.6-fold [38]. Notably, the massive outbreaks that occurred after 2015 in these two hotspots might have been triggered by climate changes that have already impacted neighboring countries [25,64,65]. Our study demonstrated that regional climate phenomena have stronger impacts on dengue transmission, echoing the recent warnings about climate change and the vulnerability to dengue transmission in areas neighboring the Mekong River [66].

In countries where dengue is endemic, mosquito activity was monitored for vector control interventions and to evaluate the effectiveness of control methods. Studies conducted in Taiwan indicated that mosquito abundance was a predictor of the dengue transmission risk [67] and that mosquito indices and climatic variables were early warning factors [68]. Herein, we found a positive association between container index and dengue transmission, suggesting that the mosquito population plays an important role in dengue fever transmission in central Vietnam. This finding is consistent with a report from the central highlands in Vietnam that showed mosquito indices and weather variables were the main risk factors for dengue transmission [69]. However, mosquito surveillance has been undertaken only monthly in Vietnam since 2015. Surveys of adult mosquitos and larvae/pupae are not always accurate due to shortage of manpower and finance. Surveys of mosquito and larval populations are presently performed only in certain regions and on a monthly basis; therefore, better survey strategies are needed to improve the quality of data while leveraging limited resources.

This preliminary analysis of dengue transmission in central Vietnam has two main limitations. First, the spatial and temporal resolutions of dengue case data and vector data were not consistent throughout the study period. Therefore, the spatial and temporal analyses and the climate models used were not in the same temporal frame. The Department of Vector Control and Border Quarantine at the Nha Trang Pasteur Institute has continued to improve the quality of its data; therefore, further analyses can be performed in the future. The second limitation is that socioeconomic characteristics, viral serotypes, and human movement were not included in the analysis. Dengue transmission results from interaction of multiple factors including vector ecology, human behavior, virus characteristics, and environmental changes. However, this is the first study to investigate the spatiotemporal pattern, the influence of climate, and vector abundance on dengue transmission in central Vietnam. A more comprehensive model is needed to forecast the risk of dengue transmission more accurately.

## 5. Conclusions

This study evaluated the effects of regional climate phenomena and mosquito population on disease transmission dynamics in central Vietnam. Faced with ongoing climate change, regional climate variations and their interactions with vector abundance and local climate conditions should be included in the dengue early warning system. Forecasting future dengue outbreaks in central Vietnam is necessary for local health policy makers to implement appropriate interventions to minimize the disease burden.

## Figures and Tables

**Table 1:** Climate variables in Da Nang and Khanh Hoa, 2014–2018.

Variable	Area	Median (IQR)	Mean (SD)	Min	Max
DTM	Da Nang	28.44 (4.7)	28.09 (3.2)	21.3	33.36
	Khanh Hoa	29.26 (4.5)	28.73 (2.8)	22.89	33.08
NTM	Da Nang	21.52 (4.6)	20.67 (3.1)	13.08	25.44
	Khanh Hoa	20.3 (4.7)	20.52 (2.2)	15.95	23.77
MTM	Da Nang	25.3 (4.2)	24.59 (2.9)	18.45	29.33
	Khanh Hoa	25.24 (3.3)	24.61 (2.4)	18.9	28.55
Rainfall	Da Nang	16.21 (48.3)	49.86 (102.2)	0	563.65
	Khanh Hoa	4.09 (12.9)	19.32 (43.4)	0	239.32

DTM—daytime temperature; IQR—interquartile range; NTM—nighttime temperature; MTM—mean temperature; SD—standard deviation.

**Table 2:** Generalized additive model (GAM) models of climate variables and dengue incidence in Da Nang and Khanh Hoa

	Model 1		Model 2		Model 3		Model 4		Model 5	
Province/Variable	Coefficient	*p*-Value	Coefficient	*p*-Value	Coefficient	*p*-Value	Coefficient	*p*-Value	Coefficient	*p*-Value
**Da Nang**										
NTM	1.293	0.06			1.443	0.076	1.001	0.0065	1.001	0.172
Rainfall (lag, 2)	2.414	0.0028	2.409	0.0033			2.265	0.0094	2.442	<0.001
IOD	2.654	0.0018	2.908	<0.001	2.377	0.022			1.489	0.018
MEI (lag, 1)	3.123	0.0043	2.929	0.0194	3.349	<0.001	2.425	0.068		
AIC	405.873		408.094		415.94		425.219		414.171	
∆AIC			2.221		10.067		19.346		8.298	
**Khanh Hoa**										
DTM	1	0.0031			1.429	0.004	1.988	0.002	1	0.0119
Rainfall	1.715	0.033	1.888	0.018			1	0.0028	1.033	0.277
IOD (lag, 1)	2.821	<0.001	2.702	<0.001	2.93	<0.001			3.011	<0.001
MEI	3.187	<0.001	3.503	<0.001	3.313	<0.001	3.063	<0.001		
AIC	333.38		339.45		337.19		356.86		370.78	
∆AIC			6.07		3.81		23.48		37.4	

AIC—Akaike’s information criterion; IOD—Indian Ocean Dipole; MEI—multivariate ENSO index; DTM—daytime temperature; NTM—nighttime temperature.

**Table 3:** GAM models of climate, mosquito index, and dengue incidence in Da Nang and Khanh Hoa

	Model 1		Model 2		Model 3		Model 4	
Province/Variable	Coefficient	*p*-Value	Coefficient	*p*-Value	Coefficient	*p*-Value	Coefficient	*p*-Value
**Da Nang**								
IOD (lag, 1)	2.676	0.0152			2.817	0.0298	2.205	0.001
MEI	3.455	0.0071	3.416	0.0243			3.178	<0.001
CI (lag, 3)	2.968	0.0094	1.955	0.0094	3.107	<0.001		
AIC	294.2421		300.8429		309.1359		411.4024	
∆AIC			6.6008		14.8938		117.1603	
**Khanh Hoa**								
IOD (lag, 1)	2.203	<0.001			1.901	<0.001	2.877	<0.001
MEI	3.188	<0.001	3.218	<0.001			3.682	<0.001
CI (lag, 1)	1	<0.001	2.865	<0.001	1	0.001		
AIC	245.8264		258.581		273.5994		343.5945	
∆AIC			12.7546		27.773		97.7681	

AIC—Akaike’s information criterion; CI—container index; IOD—Indian Ocean Dipole; MEI—multivariate ENSO index.

## Appendix A

**Table A1:** Monthly climatic variables in Da Nang and Khanh Hoa, 2014–2018

Month	Da Nang				Khanh Hoa			
	DTM	NTM	MTM	Rainfall	DTM	NTM	MTM	Rainfall
Jan	23.15	16.44	20.17	16.69	24.57	17.27	20.87	5.99
Feb	25.83	17.39	21.6	5.98	26.37	18.06	22.15	4.15
Mar	28.44	20.8	24.71	11.23	29.53	20.13	24.97	4.33
Apr	31.08	22.01	26.45	6.66	31.46	22.14	26.81	1.51
May	31.47	23.34	27.57	20.95	31.6	22.71	27.14	6.58
Jun	31.57	24.16	28.21	23.82	31.06	22.84	27.04	0.66
Jul	29.73	23.54	27.15	14.34	30.9	22.72	26.59	4.48
Aug	30.76	23.1	26.66	36.36	31.18	22.26	26.31	3.08
Sep	29.17	22.37	25.74	39.45	29.58	21.45	25.48	24.99
Oct	26.22	20.59	23.7	114.96	27.85	19.9	23.78	94.93
Nov	25.71	19.32	22.62	268.54	26.19	19.54	23.3	71.63
Dec	24	14.93	20.51	39.34	24.47	17.25	20.9	9.45

DTM—daytime temperature; NTM—nighttime temperature; MTM—mean temperature.
